# pH/thermal dual-responsive multifunctional drug delivery system for effective photoacoustic imaging-guided tumor chemo/photothermal therapy

**DOI:** 10.1063/5.0139929

**Published:** 2023-03-22

**Authors:** Jun Wang, YanYan Wu, Kai Liu, Weitao Yang, Weiwei Zeng, Xiaolong Gao, ShiYuan Liu, Bingbo Zhang

**Affiliations:** 1 Department of Radiology, Tongji Hospital, Shanghai Frontiers Science Center of Nanocatalytic Medicine, The Institute for Biomedical Engineering and Nano Science, School of Medicine, Tongji University, Shanghai 200065, China; 2 Department of Radiology, The Second Affiliated Hospital of Naval Medical University, Shanghai 200003, China; 3 Department of Radiology, Luodian Hospital, Baoshan District, Shanghai 201908, China

## Abstract

The development of a combination of chemo/photothermal therapy could overcome the limitations of single-modality therapy and enhance therapeutic efficacy. In this study, a pH/thermal dual-responsive multifunctional drug delivery system with dual-drug loading and enhanced chemo/photothermal therapy is developed based on polydopamine-coated mesoporous silica-gold nanorods (PDA-AuNRs@MSN). Nanoscale mesoporous silica-gold nanorods encapsulating doxorubicin (DOX) are designed as a core and then modified by polydopamine. The PDA shell not only conjugates with another anticancer bortezomib (Btz) to form pH-sensitive bond through boronic acid and catechol but also acts as a gatekeeper to control the release of doxorubicin and enhance the photothermal effect. Such a nanocarrier not only acts as a contrast agent for PA imaging but also serves as a therapeutic agent for enhanced chemo/photothermal therapy. The DOX and Btz could be released in an on-demand mode under near-infrared light irradiation and acid environment. The tumor size and location could be observed via PA imaging after intravenous injection into 4T1-bearing mice. Compared with AuNRs@MSN, PDA-AuNRs@MSN exhibit an increased near-infrared (NIR) absorption at 808 nm and an enhanced photothermal effect. The integrated D/B-PDA-AuNRs@MSN nanoparticles show higher cell apoptosis and enhanced tumor treatment efficacy *in vitro* and *in vivo* in comparison with single chemotherapy or photothermal therapy. Combined together, D/B-PDA-AuNRs@MSN show pH/thermal-responsive controlled-release and synergistic chemo/photothermal therapy for tumor.

## INTRODUCTION

Cancer is one of the greatest threats to human health.^1,2^ At present, chemotherapy is the most typical and conventional way for cancer treatment in clinical practice. However, single chemotherapy often results in adverse side effects and chemoresistance, which is difficult to achieve the desired therapeutic efficacy. Recently, the near-infrared (NIR) light-driven nanomaterials-mediated photothermal therapy (PTT) has attracted extensive attention due to its advantages of being noninvasive and few side effects. The mechanism of the PTT is based on the principle that the photothermal agents absorb the NIR light converting into heat, leading to cancer cell ablation and death.^3,4^ With the rapid development of nanomedicine, a wide variety of photothermal agents are developed for tumor therapy, including carbon nanomaterials,^5,6^ noble metal,^7,8^ metal sulfide,^9–11^ and organic nanomaterials.^12,13^ Among them, gold nanomaterials such as gold nanoparticles (AuNPs),^14,15^ gold nanorods (AuNRs),^16,17^ gold nanocages (AuNCs),^18,19^ gold nanostars,^20,21^ gold nanoshell,^22,23^ and gold nanoflowers^24,25^ have been extensively explored as photothermal agents for cancer therapy.

Among these gold nanomaterials, AuNRs have been widely used in the field of biomedicine due to the tunability of longitudinal surface plasmon resonance (LSPR), high yield, ease of synthesizing small size, and excellent stability.^26^ However, PTT alone could not kill cancer cells completely due to the local heterogeneous distribution of heat in tumors, light scattering, and absorption.^27^ In addition, the energy of the light was gradually decreased with the depth of penetration into the tissues.^28^ Compared with the single therapy mode, synergistic combination therapy, such as chemo-photothermal therapy, is considered as an effective strategy to enhance therapeutic efficiency because this strategy has the advantages of reducing the negative effects and overcoming the drug resistance of tumor cells. However, the treatment systems based on AuNRs hold the disadvantages of loading one antitumor drug or a low therapeutic efficiency.^29,30^ Therefore, the construction of the AuNRs theranostic system to achieve imaging-guided dual-drug delivery, controllable-responsive release, and enhanced chemo/photothermal therapy for tumor still remains a great challenge.

Polydopamine (PDA) is a kind of biomimetic material of mussel, which can be obtained by self-polymerization of dopamine in weak alkaline environment.^31^ PDA has many excellent properties, such as simple preparation, good biocompatibility, and excellent photothermal properties.^32^ The structure of PDA contains a large amount of catechol and primary and secondary amines, so that PDA can be adsorbed on the surface of most substances to form a layer of polyamine film.^33^ In addition, PDA adsorbed on the surface of the materials can act as a reaction “bridge” to further react with reagents containing nucleophilic groups through Michael addition or Schiff base reaction, thereby introducing other functional groups or substances on the surface of the materials.^34^ Finally, PDA has an excellent near-infrared photothermal conversion function, which can be used for tumor photothermal therapy. Based on the intrinsic properties of PDA, PDA-modified nanoparticles not only deliver chemotherapeutic drugs but also enhance the photothermal therapy.

In this study, we developed a double-drug loading and pH/thermal dual-sensitive drug delivery system for PA imaging-guided multi-modal cancer therapy based on polydopamine-coated mesoporous silica-AuNRs. In this drug delivery system, nanoscale mesoporous silica coated AuNRs encapsulating doxorubicin hydrochloric acid (DOX) was designed as a core. Polydopamine (PDA) was deposited on the surface of the mesoporous silica@AuNRs by oxidative self-polymerization for enhanced PTT and controlled drug release as gatekeepers. Another antitumor drug bortezomib (Btz) was combined to PDA through boronic acid of Btz and catechol of PDA conjugation. D/B-PDA-AuNRs@MSN can not only be a PA imaging agent but also serve as an enhanced chemo/photothermal combined therapy. Such nanoparticles could generate PA signal and an enhanced photothermal effect under 808 nm laser irradiation. Meanwhile, the loaded drug DOX/Btz could effectively release by pH in the tumor microenvironment and the NIR-induced photothermal effect of D/B-PDA-AuNRs@MSN, resulting in synergistically enhancing tumor therapy efficacy.

## RESULTS AND DISCUSSION

### Synthesis and characterizations of D/B-PDA-AuNRs@MSN

**Scheme 1.**
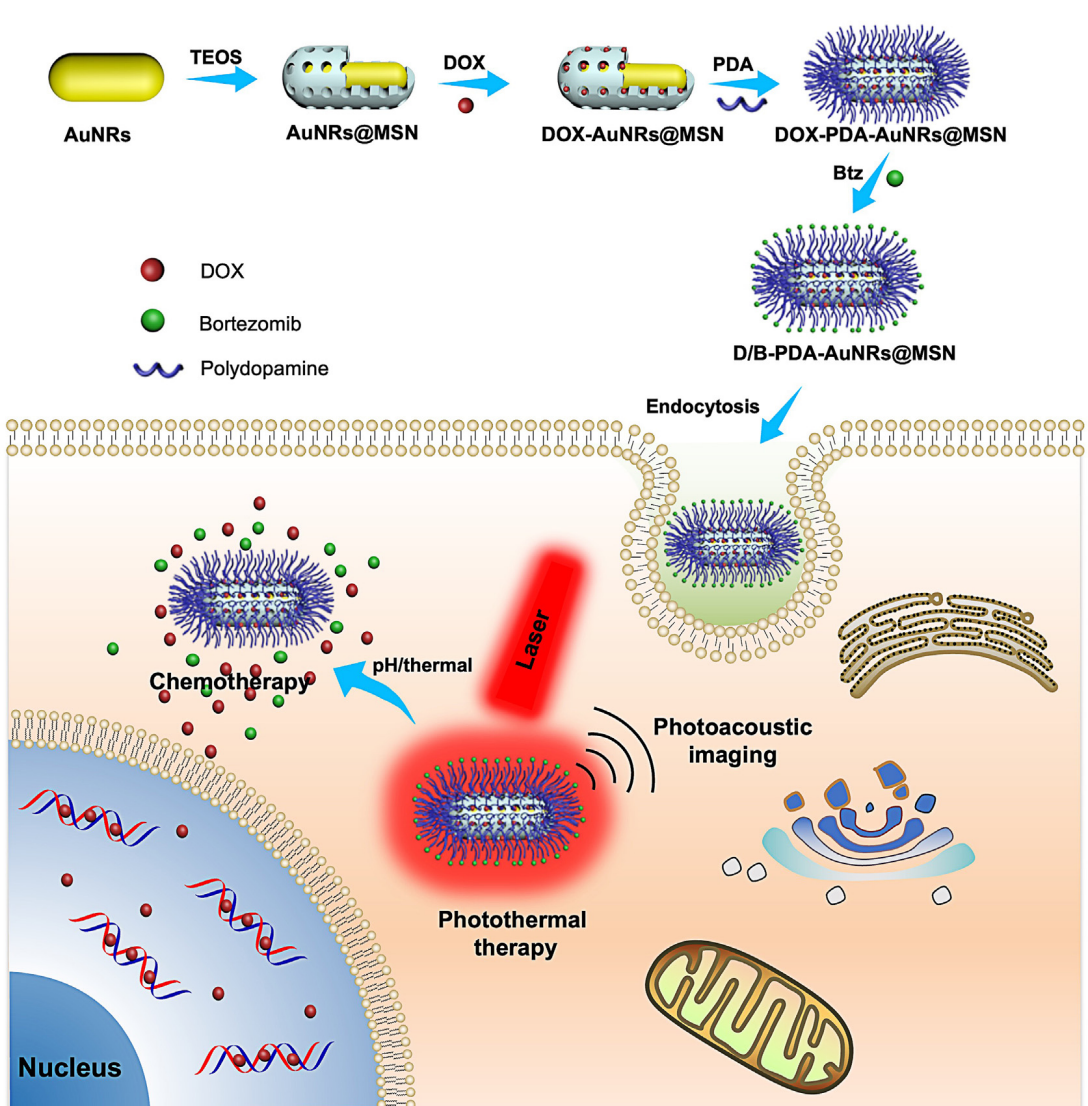
Illustration of the synthesis of D/B-PDA-AuNRs@MSN and its applications for enhanced chemo/photothermal combined therapy and controlled drug release.

**FIG. 1.**
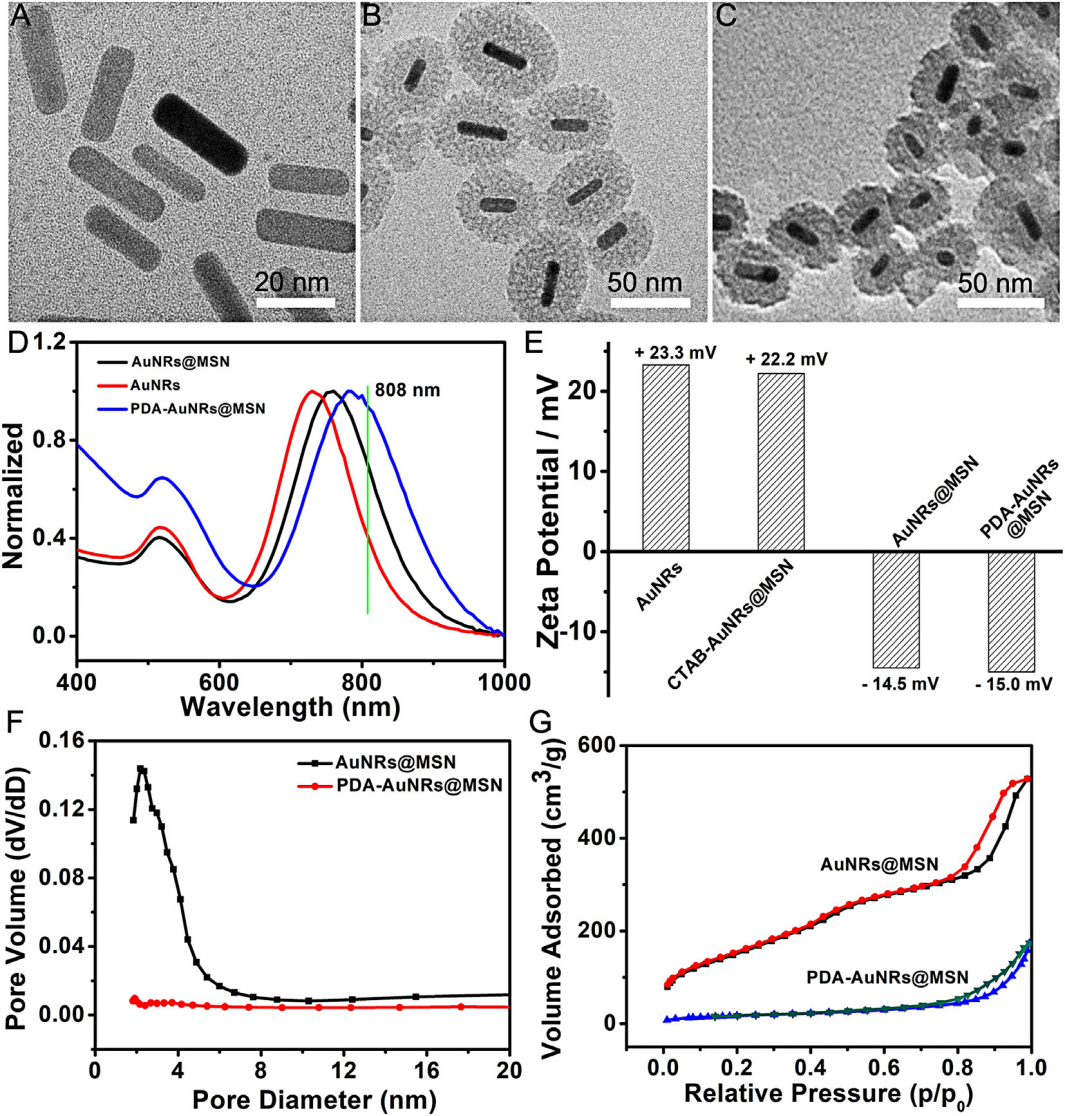
The TEM images of the AuNRs (a), AuNRs@MSN (b), and PDA-AuNRs@MSN (c); the UV-vis spectrum of the AuNRs, AuNRs@MSN, and PDA-AuNRs@MSN (d); the changes of the zeta potential after each modification (e); nitrogen adsorption–desorption isotherms (f) and pore size distributions (g) of the as-synthesized AuNRs@MSN and PDA-AuNRs@MSN.

The synthesis of D/B-PDA-AuNRs@MSN was shown in Scheme 1. First, a one-pot seedless-mediated growth method was adopted to synthesize CTAB-stabilized AuNRs. Transmission electron microscopy (TEM) images show that the AuNRs are about 25 nm in length and 8 nm in width [Fig. 1(a)] and longitudinal surface plasmon resonance (LSPR) peak of 730 nm [Fig. 1(d)]. Then, a mesoporous silica shell layer coating on the surface of the AuNRs by the sol–gel method is observed, and the thickness of the silica shell is about 20 nm [Fig. 1(b)]. After coating, the LSPR peak was found to shift to 757 nm due to the surface refractive index changes [Fig. 1(d)]. The anticancer drug DOX was loaded into the pores of the AuNRs@MSN by diffusing prior to the PDA deposition. Subsequently, the DOX-AuNRs@MSN was incubated with dopamine hydrochloride in Tris-HCl (pH 8.5, 10 mM) solution to form polydopamine layer. After coating with PDA, a block layer on the surface of the AuNRs@MSN can be observed [Fig. 1(c)], and the peak of the LSPR has a further red shift to 783 nm [Fig. 1(d)]. The hydrodynamic diameter of the PDA-AuNRs@MSN is about 40 nm (Fig. S1). The loading of another anticancer drug Btz on the DOX-PDA-AuNRs@MSN was conducted through the conjugation between boronic acid of Btz and catechol of PDA.

Each modification of the AuNRs is confirmed by FT-IR, zeta potential, and N_2_ absorption–adsorption. FT-IR spectrum of CTAB-AuNRs@MSN shows the characteristic C–H stretching vibrations at 2922 and 2856 cm^−1^ and C–H deformation vibration around 1480 cm^−1^.^35^ These C–H peaks disappear after removing CTAB. Compared to AuNRs@MSN, PDA-AuNRs@MSN displays an absorption band of 1290 cm^−1^, which is assigned to the stretching vibration of C–O and primary amine vibration from PDA, which indicates the PDA layer is successfully coated on the surface of the AuNRs@MSN (Fig. S2).^36^ Due to the existence of the CTAB, the AuNRs and CTAB-AuNRs@MSN exhibit a zeta potential of +23.3 and +22.2 mV, respectively. After removing the template of CTAB, the potential of AuNRs@MSN has a negative value of −14.5 mV. Moreover, after the AuNRs@MSN are coated with the PDA shell, the potential of the obtained nanoparticles is still −15.0 mV because of the catechol groups on the surface of the PDA-AuNRs@MSN nanoparticles [Fig. 1(e)].^36^ Figures 1(f) and 1(g) show the nitrogen adsorption–desorption isotherms and the pore size distribution diagram of the prepared AuNRs@MSN and PDA-AuNRs@MSN nanoparticles. For the AuNRs@MSN, the BET surface area is 564.76 m^2^ g^−1^, the pore volume is 0.82 cm^3^ g^−1^, and the pore size is about 2.33 nm. After depositing the PDA shell, the BET specific surface area, pore size, and pore volume of the PDA-AuNRs@MSN nanoparticles are smaller than the AuNRs@MSN nanoparticles and are 62.35 m^2^ g^−1^, 1.89 nm, and 0.2 cm^3^ g^−1^, respectively. This further suggests that the PDA shell has been successfully modified on the surface of the AuNRs@MSN nanoparticles.

### The photothermal effect of PDA-AuNRs@MSN and drug release *in vitro*

**FIG. 2.**
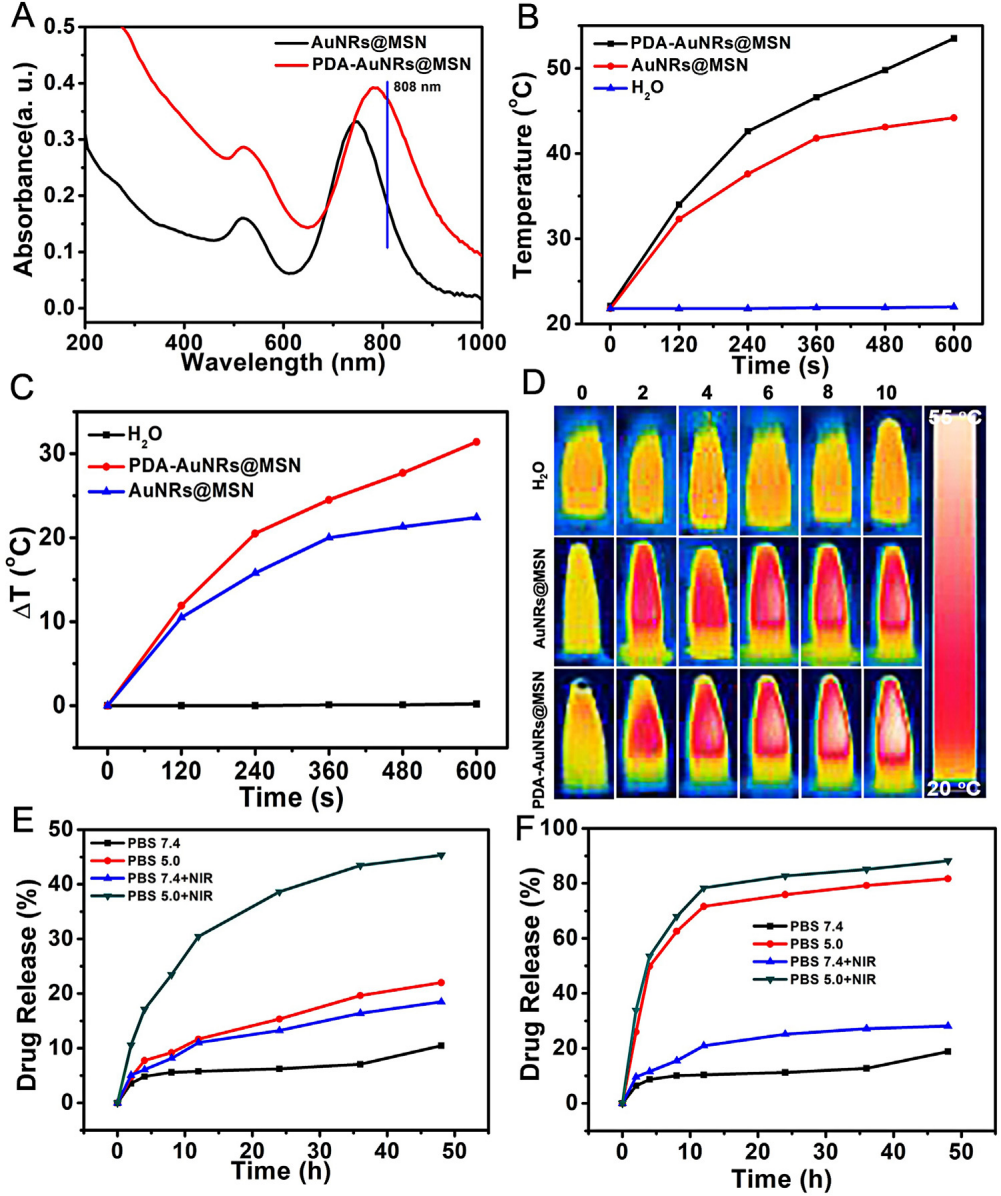
The UV-vis absorption spectra of PDA-AuNRs@MSN and AuNRs@MSN aqueous solution at the same Au concentration (20 *μ*g/ml) (a); temperature elevation of PDA-AuNRs@MSN and AuNRs@MSN under NIR laser irradiation (808 nm, 1 W/cm^2^, and 10 min) (b); temperature changes *vs* PDA-AuNRs@MSN and AuNRs@MSN (c); the infrared thermal images of PDA-AuNRs@MSN and AuNRs@MSN at the same different Au concentration under NIR laser irradiation (808 nm, 1 W/cm^2^, and 10 min) (d); the cumulative drug release profiles of the DOX (e) and Btz (f) from D/B-PDA-AuNRs@MSN in PBS at pH values of 7.4 and 5.0 with or without NIR irradiation, respectively.

In order to prove the deposition of the PDA layer could enhance the photothermal effect, the UV-vis absorption spectra of PDA-AuNRs@MSN and AuNRs@MSN were measured. As shown in [Fig. 2(a)], compared with AuNRs@MSN, the PDA-AuNRs@MSN exhibited a remarkable increase at 808 nm at the same Au concentration.

Then, the photothermal of the PDA-AuNRs@MSN and AuNRs@MSN by 808 nm laser irradiation at a power intensity of 1.0 W cm^−2^ for 10 min was investigated. The pure water shows little temperature change, which is increased by only 0.2 °C. The temperature of PDA-AuNRs@MSN is found increased by 31.4 °C and significantly higher than that of AuNRs@MSN (22.4 °C) under the same condition. The results demonstrate that the enhanced photothermal effect of the PDA-AuNRs@MSN can be attributed to the PDA shell, which increases NIR absorption at 808 nm [Fig. 2(b)]. Meanwhile, the photothermal conversion efficiency of the AuNRs@MSN and PDA-AuNRs@MSN is evaluated. According to the formula of the photothermal conversion (PTC) efficiency, the PTC of the PDA-AuNRs@MSN is calculated to be 34.71%, which is higher than that of AuNRs@MSN (18.37%). The above results indicate that the enhanced photothermal effect is attributed to the PTC efficiency (Fig. S3). Then, we investigated the photothermal effect of different concentrations of PDA-AuNRs@MSN (Au = 10, 20, 40, and 60 *μ*g/ml). We found the temperature increased along with the concentration of the PDA-AuNRs@MSN under the laser continuous irradiation for 10 min (Fig. S4).

Double anticancer drugs, DOX and Btz, were loaded into the nanoparticles through two different mechanisms. DOX loading was achieved by physical absorption, while Btz was realized by covalent conjugation. The UV-vis spectrum confirms both two anticancer drugs were successfully loaded in the PDA-AuNRs@MSN (Fig. S5). The loading contents of DOX and Btz in PDA-AuNRs@MSN were measured to be 115 and 6.1 mg/g by calculating the absorbance of the supernatant according to the DOX and Btz standard curve (Fig. S6), respectively. Figures 2(e) and 2(f) show the drug release profiles of DOX and Btz in different pH solutions with or without the laser irradiation. The release of both anticancer drugs release is pH-dependent. For DOX, the drug release rate is much less at pH 7.4 than that at pH 5.0 because of the PDA layer might be partially peeled from the surface of the nanoparticles at pH 5.0. For Btz, about 81.7% of the Btz is released at pH 5.0, while only 18.8% of the Btz is released at pH 7.4. This difference is attributed to the pH dependence of cleavage of the boronic ester bond. The drug release rates of DOX and Btz are obviously faster under NIR irradiation at different pH values. The release of Btz from the nanoparticles can be increased to 88.2% under laser irradiation and acidic condition. This condition can facilitate the cleavage of the boronic ester for drug release.^37^ While for the release rate of DOX, it is a slightly increase, which can be ascribed to the heat that accelerates the DOX molecules movement at pH 5.0. These results indicate the release of DOX and Btz is sensitive to pH and heat-dependent [Figs. 2(e) and 2(f)].

### *In vitro* cytotoxicity and cell uptake

**FIG. 3.**
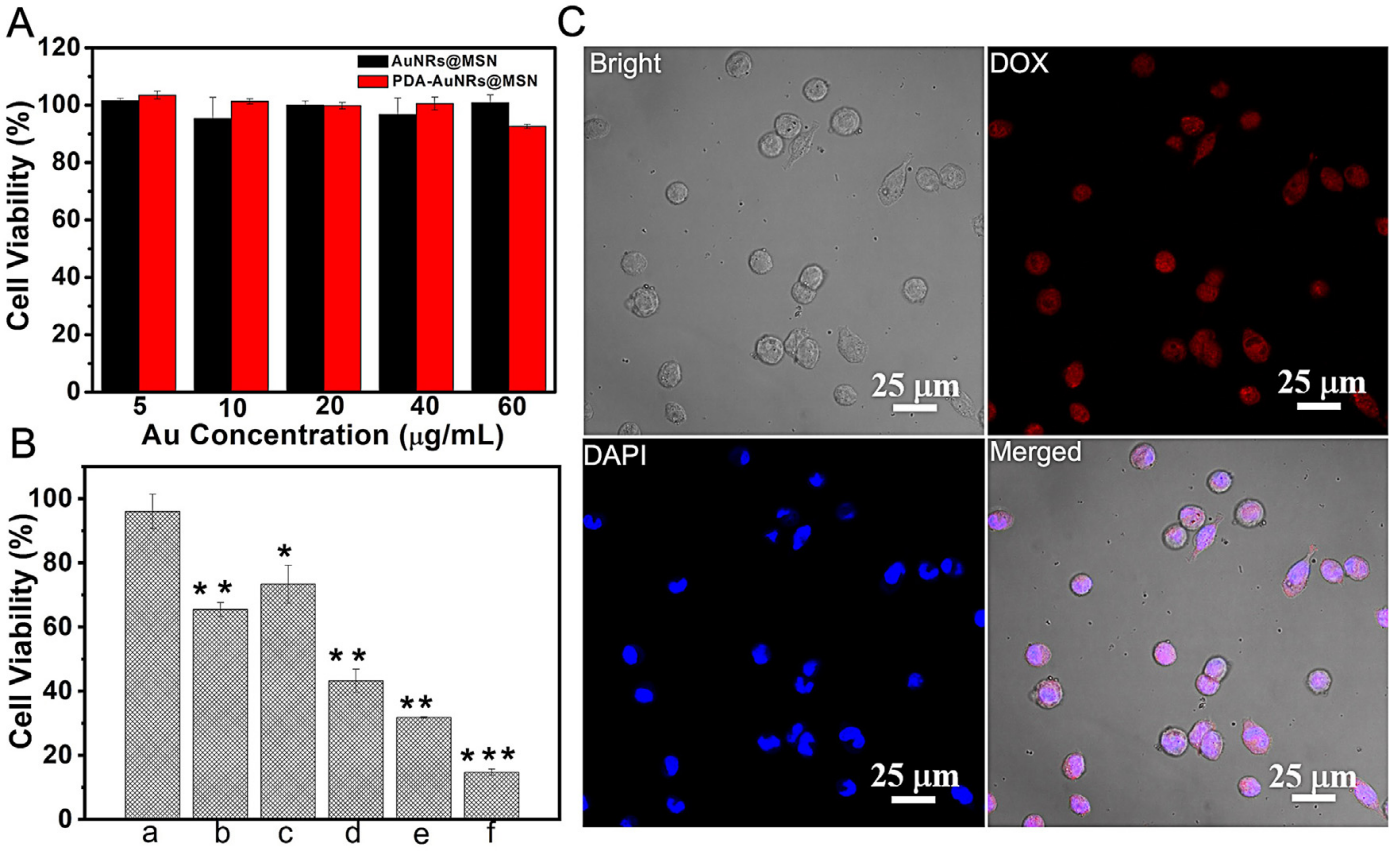
The cell viabilities of the 4T1 cells treated with AuNRs@MSN and PDA-AuNRs@MSN at different concentrations for 24 h (a); the cell viabilities of 4T1 cells treated with different treatments: PBS+NIR (a), free DOX/Btz (D/B) (b), D/B-PDA-AuNRs@MSN (c), AuNRs@MSN+NIR (d), PDA-AuNRs@MSN+NIR (e), and D/B-PDA-AuNRs@MSN+NIR (f) (DOX concentration: 2 *μ*g/ml, Btz concentration: 0.1 *μ*g/ml) (b); the confocal microscopic images of 4T1 cells incubated with D/B-PDA-AuNRs@MSN for 4 h at 37 °C (DOX concentration = 2 *μ*g/ml). The nuclei were stained by DAPI (c). 0.01 <^*^p < 0.05, 0.001 <^**^p < 0.01, and ^***^p < 0.001.

The cytotoxicity of AuNRs@MSN and PDA-AuNRs@MSN was evaluated by CCK-8 assay. The cell viabilities of the 4T1 cells are over 90% after incubation with AuNRs@MSN and PDA-AuNRs@MSN even at 60 *μ*g/ml of Au concentration, suggesting that the nanoparticles are biocompatibility [Fig. 3(a)]. In order to evaluate the combined therapeutic effect, 4T1 cells were treated with several groups with or without NIR laser irradiation. As shown in [Fig. 3(b)], compared with free D/B, D/B-PDA-AuNRs@MSN shows higher cell cytotoxicity. This is because the drug release is inhibited by the PDA layer. However, upon laser irradiation, D/B-PDA-AuNRs@MSN shows much higher cell killing capability than that of free D/B drugs, AuNRs@MSN, and PDA-AuNRs@MSN. To be specific, PBS+laser treatment shows very little killing effect on cells due to the low NIR light absorption by endogenous cytochromes. Owing to the enhanced photothermal effect of gold nanorod, ∼32.1% of cells were found killed by AuNRs@MSN and ∼69.4% of cells were killed by PDA-AuNRs@MSN in the presence of NIR irradiation. Nevertheless, D/B-PDA-AuNRs@MSN coupled with NIR irradiation exhibited the highest cytotoxicity, which further confirmed D/B-PDA-AuNRs@MSN exhibited an enhanced chemo/photothermal therapy when combining with PDA, DOX, and Btz into a single nanoplatform.

The intracellular uptake of the D/B-PDA-AuNRs@MSN in 4T1 cells was investigated using confocal laser scanning microscopy. After 4 h incubation, red signals were observed in the cell nucleus and cytoplasm, which indicates that the D/B-PDA-AuNRs@MSN could be effectively phagocytized by cells, and the loaded DOX could be released from the D/B-PDA-AuNRs@MSN into the cell nucleus [Fig. 3(c)].

### *In vivo* photoacoustic imaging and photothermal-chemo therapy

Prior to *in vivo* application, we first studied the potential *in vivo* toxicity of the PDA-AuNRs@MSN. HE staining results demonstrate that the main organs (heart, liver, spleen, lung, kidney, and intestines) have no inflammation or abnormality after 15 days compared with the control group, showing the PDA-AuNRs@MSN could be applied to *in vivo* biomedical application (Fig. S7).

**FIG. 4.**
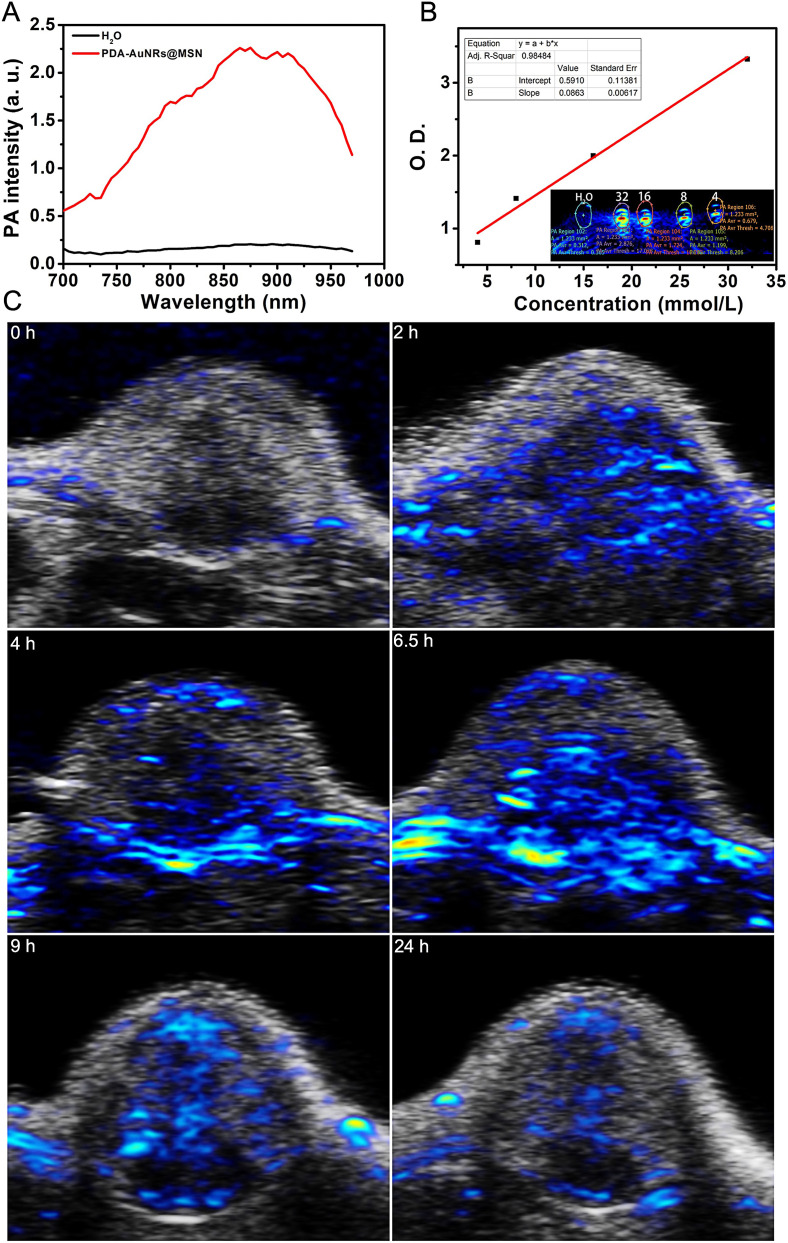
Determination of the optimal excitation wavelength of photoacoustic imaging (a); the linear relationship between PA signal intensity and concentration of PDA-AuNRs@MSN, and the inset image is PA imaging phantoms (b); *in vivo* photoacoustic images of the 4T1-tumor-bearing mouse with intravenous injection of PDA-AuNRs@MSN at different time points (c). Note: the excitation wavelength is 875 nm.

Due to high NIR absorption, we further investigated the PDA-AuNRs@MSN for *in vivo* PA imaging, which is noninvasive biomedical imaging with high imaging depth and spatial resolution.^38^ The optimal photoacoustic excitation wavelength *in vitro* was found to be 875 nm [Fig. 4(a)], and the PA intensity of the PDA-AuNRs@MSN becomes stronger with the increase in the concentration gradually and exhibits a certain linear correlation [Fig. 4(b)]. Furthermore, we conducted *in vivo* PA imaging of the PDA-AuNRs@MSN on 4T1 tumor-bearing mice. As shown in Figs. 4(c) and S8, a weak PA signal is observed before injecting the PDA-AuNRs@MSN via tail vein. The PA signal can be observed in the tumor site at 2 h, and the intensity is gradually enhanced along with the time and reaches the maximum within 6.5 h, demonstrating the accumulation of PDA-AuNRs@MSN in tumor and could be a good PA imaging contrast agent *in vivo*.

**FIG. 5.**
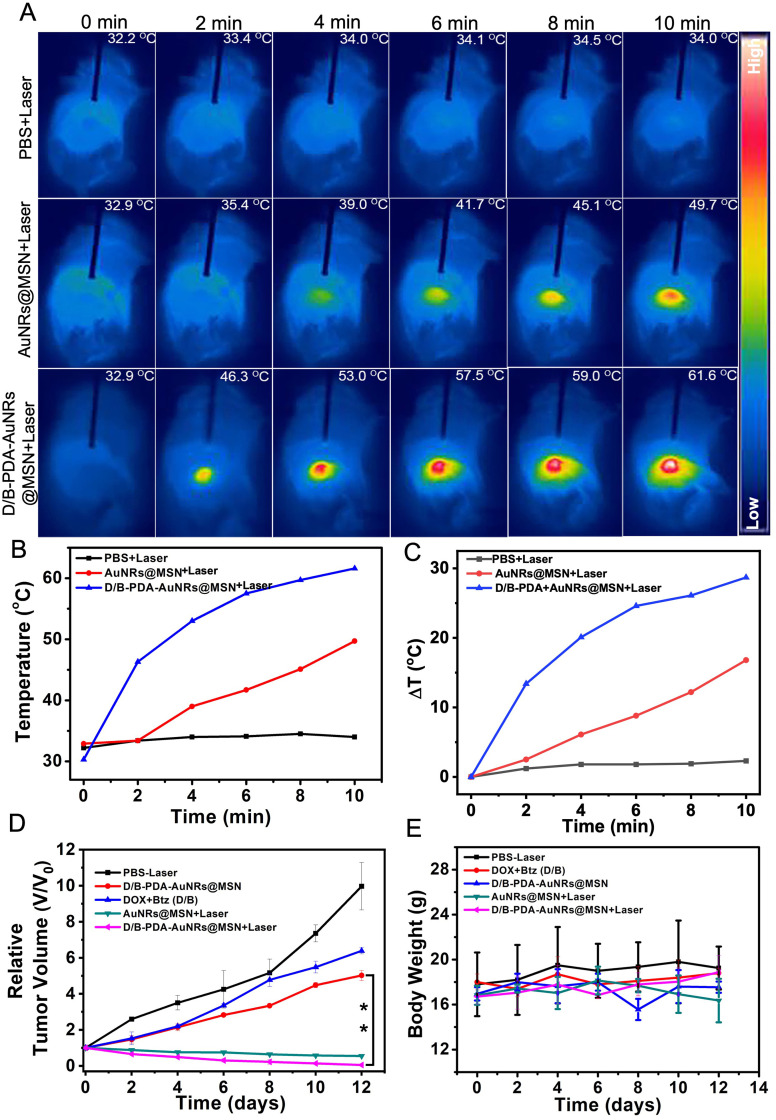
The infrared thermal images of 4T1-tumor-bearing mice with intravenous injection of PBS (200 *μ*l), AuNRs@MSN (200 *μ*l, 32 *μ*mol/l), and D/B-PDA-AuNRs@MSN (200 *μ*l, 32 *μ*mol/l) and then irradiated with an 808 nm NIR laser (1.0 W/cm^2^) for 10 min (A); the changes of the temperature at the tumor site from different treatment groups (b) and (c); tumor growth (d) and body weight (e) curves after different treatments. Scale bar is 100 *μ*m. 0.01 <^*^p < 0.05, 0.001 <^**^p < 0.01, and ^***^p < 0.001.

We finally utilized the D/B-PDA-AuNRs@MSN for tumor photothermal-chemo therapy. The 4T1 tumor-bearing mice were randomly divided into five groups: PBS+Laser, DOX+Btz, D/B-PDA-AuNRs@MSN, AuNRs@MSN+Laser, and D/B-PDA-AuNRs@MSN+Laser. Upon laser irradiation for 10 min, the tumor temperature rapidly reaches to 61.6 °C, which would be high enough to kill the tumor cells. In contrast, the tumor temperature treated with AuNRs@MSN and PBS raises by 49.7 and 34.0 °C, respectively [Figs. 5(a)–5(c)]. Compared to the AuNRs@MSN group, the tumor growth of the D/B-PDA-AuNRs@MSN is significantly inhibited after irradiation. It should be noted that PBS+laser has no treatment effect on tumor since the temperature is not high enough under laser irradiation. Free drugs are rapidly metabolized *in vivo*. The D/B-PDA-AuNRs@MSN can be accumulated in the tumor site through the enhanced permeability and retention (EPR) effect, and, thus, the inhibitory effect of D/B-PDA-AuNRs@MSN on tumor is better than D/B alone [Fig. 5(d)]. Meanwhile, no obvious body weight loss is observed during the whole treatment period [Fig. 5(e)].

The tumor of each group was stripped for HE analysis to evaluate the therapeutic efficiency after the treatment period. The D/B-PDA-AuNRs@MSN group shows significant cancer cell damage than either photothermal therapy or chemotherapy alone (Fig. S9). Taken together, the D/B-PDA-AuNRs@MSN could serve as a theranostic probe for tumor imaging-guided therapy.

## CONCLUSION

In summary, we designed a multifunctional drug delivery system based on D/B-PDA-AuNRs@MSN for tumor PA imaging-guided chemo-photothermal therapy, which combines double-drug loading and pH/thermal dual-responsive drug release. The presence of the PDA layer not only loads the anticancer drug and controls the drug release but also enhances the photothermal effect. The *in vitro* and *in vivo* experiments show D/B-PDA-AuNRs@MSN can be effectively internalized by the cancer cells, and particularly, it exerts high-quality PA imaging-guided chemo and photothermal therapy in tumor. Thus, the developed multifunctional D/B-PDA-AuNRs@MSN theranostic probe could serve as an effective probe for the treatment of cancers.

## METHODS

### Materials

Tetraethylorthosilicate (TEOS, 99%) was purchased from Sigma-Aldrich. Cetyltrimethylammonium bromide (CTAB), ascorbic acid (AA), ethanol, and hydrochloric acid were acquired from Sinopharm Chemical Reagent Co., Ltd. Sodium borohydride (NaBH_4_), 4, 6-diamidino-2-phenylindole (DAPI), and sodium hydroxide were purchased from Aladdin (Shanghai, China). Silver nitrate (AgNO_3_) and dopamine hydrochloride were purchased from Alfa Aesar. Tetrachloroauric acid (HAuCl_4_·3H_2_O) was purchased from Huawei Chemical Reagent Co., Ltd. Doxorubicin hydrochloride (DOX·HCl, 98%) and bortezomib were obtained from Dalian Meloney Biotechnology Co., Ltd (Dalian, China). Millipore water with 18.2 MΩ was used in the experiment.

### Synthesis of AuNRs

The AuNRs were prepared using a seedless method with slight modifications.^39,40^ Briefly, CTAB solution (30 ml, 0.2 M) was added to 30 ml of 1.0 mM HAuCl_4_, followed by the addition of 1.8 ml of 4 mM AgNO_3_ and 72 *μ*l of HCl (37%). Next, 450 *μ*l of 85.8 mM AA was added and gently swirled as the solution became colorless. Finally, 45 *μ*l of 10 mM NaBH_4_ was rapidly injected. The resulting solution was kept for 6 h at 30 °C.

### Preparation of AuNRs with mesoporous silica shell (AuNRs@MSN)

AuNRs@MSN were synthesized according to the previous report.^41,42^ To remove excess CTAB from AuNRs, 30 ml of the as-synthesized AuNRs was centrifuged at 16 000 rpm for 30 min. The precipitate was redispersed in 30 ml of Milli-Q water, and 300 *μ*l of 0.1 M NaOH solution was added upon stirring. Then, three injections of 90 *μ*l of 20% TEOS in methanol solution were added into the above solution at 30 min intervals. The mixture was stirred for 24 h at 30 °C. The AuNRs@MSN were separated by centrifugation. The precipitate was refluxed with 20 ml of 10 mg/ml NH_4_NO_3_-ethanol solution under 60 °C for 12 h to extract the surfactant template CTAB. The final product was collected by centrifugation at 16 000 rpm for 30 min and washed with ethanol three times. The as-synthesized solid was dried in the lyophilizer.

### Synthesis of PDA coated DOX-AuNRs@MSN (PDA-DOX-AuNRs@MSN)

The PDA-DOX-AuNRs@MSN were synthesized according to the literatures.^43,44^ AuNRs@MSN (50 mg) was added to the DOX solution (1 mg/ml, 5 ml) and stirred in the dark at the room temperature for 24 h. The product was acquired by centrifugation and washed with de-ionized water until the supernatant became colorless. The DOX-AuNRs@MSN (50 mg) nanoparticles were suspended in the 50 ml of Tris–HCl buffer solution (pH 8.5, 10 mM). Then, dopamine hydrochloride (25 mg) was added and stirred in the dark at room temperature for 24 h. The PDA-DOX-AuNRs@MSN were collected by centrifugation and washed with de-ionized water several times to remove the unpolymerized dopamine. PDA-AuNRs@MSN were synthesized according to the same procedure without adding the DOX. The loading efficiency (LE%) of DOX on AuNRs@MSN was calculated using the following formula: LE% = (M_DOX1_-M_DOX2_)/M_DOX1_, where M_DOX1_ is the added DOX content and M_DOX2_ is the supernatant DOX content.

### Synthesis of DOX/Btz-PDA-AuNRs@MSN (D/B-PDA-AuNRs@MSN)

Btz were conjugated to the PDA-DOX-AuNRs@MSN according to the reported literatures.^37,45^ The PDA-DOX-AuNRs@MSN were dispersed in 10 ml of dimethylsulfoxide (DMSO)-de-ionized water (1:10, v/v) solution containing 5 mg of Btz. The mixture was stirred for 24 h at room temperature. The products were separated by centrifugation and washed with de-ionized water. The loading efficiency (LE%) of Btz on DOX**-**PDA-AuNRs@MSN was calculated using the following formula: LE% = (M_Btz1_-M_Btz 2_)/M_Btz1_, where M_Btz1_ is the added Btz content and M_Btz2_ is the supernatant Btz content.

### *In vitro* drug release

D/B-PDA-AuNRs@MSN were dispersed in 2 ml of buffer solutions (pH 5.0 and 7.4). The dispersion solution was then transferred into a dialysis bag (molecular weight cut off = 8000–14 000 kDa) and placed in 100 ml of PBS buffer solution at 37 °C with or without 808 nm light irradiation and shaked at 150 rpm. At timed intervals, 3 ml of solution was withdrawn from the solution. The released DOX and Btz were analyzed by UV-vis spectrum. The volume of the release medium was kept constant by adding 3 ml of fresh medium after each sampling.

### *In vitro* cytotoxicity

The cell viability was determined by CCK-8 assay. 4T1 cells were seeded into a 96-well plate at a density of 1 × 10^4^ cells and cultured at 5% CO_2_ and 37 °C for 24 h. Different concentrations of AuNRs@MSN, PDA-AuNRs@MSN, free DOX+Btz, and D/B-PDA-AuNRs@MSN were added to the medium, and the cells were incubated at 5% CO_2_ and 37 °C for 24 h. In order to evaluate PTT efficacy, PBS, AuNRs@MSN, PDA-AuNRs@MSN, and D/B-PDA-AuNRs@MSN were cultured with 12 h before the cells were irradiated with 808 nm laser (1 W/cm^2^) for 5 min. The cells were further incubated for another 12 h. The cell viability was calculated by measuring the absorbance value at 450 nm.

### *In vitro* cellular uptake

4T1 cells were seeded into the confocal dish and cultured for 24 h. The D/B-PDA-AuNRs@MSN solution (DOX concentration= 2 *μ*g/ml and Btz concentration = 0.1 *μ*g/ml) was added and cultured with cells for 4 h. Then, the cells were washed with PBS solution and fixed with 4% formaldehyde for 10 min. After that, the cells were washed with PBS solution several times to remove excess formaldehyde. The cell nuclei was stained by DAPI. The fluorescence images were observed under confocal laser scanning microscopy (CLSM).

### Temperature measurement *in vitro*

The aqueous of AuNRs@MSN and PDA-AuNRs@MSN containing the same Au concentration (20 *μ*g/ml) was added into 0.5 ml centrifuge tube and irradiated by 808 nm laser at a power density of 1 W/cm^2^ for 600 s. For the control group, 0.5 ml of de-ionized water was also irradiated under the same condition. To investigate different concentrations of the PDA-AuNRs@MSN photothermal effect, the as-prepared PDA-AuNRs@MSN was diluted to different concentrations (Au = 10, 20, 40, and 60 *μ*g/ml), and 0.5 ml sample solution was added into the centrifuge tube and was irradiated by 808 nm laser (1 W/cm^2^) for 600 s. For the control group, 0.5 ml of de-ionized water was also irradiated under the same condition. A thermal imager was used to measure the temperature changes and obtain the infrared thermal images.

### *In vivo* biosafety analysis

Female BALB/c mice (4 weeks) were purchased from SLAC Laboratory Animal Co, Ltd. (Shanghai), and the animal procedures were complied with the guidelines of the Institutional Animal Care and Use Committee of Tongji University. Female BALB/c mice (4 weeks) were treated by the PDA-AuNRs@MSN (Au = 32 *μ*mol/l) through tail vein injection. The control group was injected with PBS pH 7.4 solution at the same volume. The main organs (heart, liver, spleen, lung, kidney, and intestines) were collected after 15 days and were stained with hematoxylin and eosin (H&E) for histological analysis.

### *In vivo* photothermal treatment

4T1 cells (2 × 10^6^ cells in PBS pH 7.4 buffer solution) were injected subcutaneously into the flank of the right fore leg of the female BALB/c mice (4 weeks). When the tumor volume reached 80 mm^3^, the mice were randomly divided into five groups (n = 3 for each group). The mice were treated via the tail vein with 100 *μ*l solution of PBS+Laser, DOX+Btz, D/B-PDA-AuNRs@MSN, AuNRs@MSN+Laser, and D/B-PDA-AuNRs@MSN+Laser. After 6 h, the mice were irradiated by 808 nm laser (1 W/cm^2^) for 600 s for laser treating groups. The tumor volume was measured by a digital caliper every 2 days. The tumor volume = length × width^2^/2.

### *In vivo* photoacoustic (PA) imaging of tumor

4T1 cells (2 × 10^6^ cells in PBS pH 7.4 buffer solution) were injected subcutaneously into the flank of the right fore leg of the female BALB/c mice (4 weeks). When the tumor volume reached 80 mm^3^, the PDA-AuNRs@MSN (Au = 32 *μ*mol/l) was intravenously injected into the mice. The PA imaging of the tumor site at different time points (0, 2, 4, 6.5, 9, and 24 h) was scanned using the Vevo LAZR system. The excitation wavelength is 875 nm.

### Characterizations

Transmission electron microscopy (TEM) was conducted on JEM-2100 operating at 200 kV. Zeta potentials were measured on a zeta potential analyzer (Zetasizer Nano ZS90, Malvern). The UV-vis spectrum of the sample was measured with a Cary 50 spectrophotometer (Varian). The surface area, pore size, and pore volume were determined by N_2_ adsorption–desorption isotherms obtained at 77 K on a Quantachrome Autosorb-1 (USA). The sample was outgassed at 10^−3 ^Torr and 60 °C for approximately 6 h prior to the adsorption experiment. The PA imaging was conducted on the Vevo LAZR system (FujiFilm VisualSonics Inc., America). Fourier Transform Infrared Spectroscopy (FTIR) was measured on a SHIMADZU IR prestige-21 spectrometer. Cell imaging was conducted on TCS SP5 confocal laser scanning microscope (Leica, Germany).

### Statistical analysis

The results were expressed as mean ± standard deviation through at least three experiments. Statistical analysis of data was performed using the GraphPad Prism software. p < 0.05 was considered as statistically significant. 0.01 < ^*^p < 0.05, 0.001 <^**^p < 0.01, and ^***^p < 0.001.

## SUPPLEMENTARY MATERIAL

See the supplementary material for the PDA-AuNRs@MSN characterization, the standard curve of DOX and Btz, the quantitative results of the PA signals, and H&E-staining.

## Data Availability

The data that support the findings of this study are available within the article and its supplementary material.
